# Cueing musical emotions: An empirical analysis of 24-piece sets by Bach and Chopin documents parallels with emotional speech

**DOI:** 10.3389/fpsyg.2015.01419

**Published:** 2015-11-02

**Authors:** Matthew Poon, Michael Schutz

**Affiliations:** Music, Acoustics, Perception and Learning Lab, McMaster Institute for Music and the Mind, School of the Arts, McMaster University, HamiltonON, Canada

**Keywords:** pitch height, timing, music and language, speech perception, music cognition, corpus analysis

## Abstract

Acoustic cues such as pitch height and timing are effective at communicating emotion in both music and speech. Numerous experiments altering musical passages have shown that higher and faster melodies generally sound “happier” than lower and slower melodies, findings consistent with corpus analyses of emotional speech. However, equivalent corpus analyses of complex time-varying cues in music are less common, due in part to the challenges of assembling an appropriate corpus. Here, we describe a novel, score-based exploration of the use of pitch height and timing in a set of “balanced” major and minor key compositions. Our analysis included all 24 Preludes and 24 Fugues from Bach’s *Well-Tempered Clavier* (book 1), as well as all 24 of Chopin’s *Preludes* for piano. These three sets are balanced with respect to both modality (major/minor) and key chroma (“A,” “B,” “C,” etc.). Consistent with predictions derived from speech, we found major-key (nominally “happy”) pieces to be two semitones higher in pitch height and 29% faster than minor-key (nominally “sad”) pieces. This demonstrates that our balanced corpus of major and minor key pieces uses low-level acoustic cues for emotion in a manner consistent with speech. A series of *post hoc* analyses illustrate interesting trade-offs, with sets featuring greater emphasis on timing distinctions between modalities exhibiting the least pitch distinction, and vice-versa. We discuss these findings in the broader context of speech-music research, as well as recent scholarship exploring the historical evolution of cue use in Western music.

## Introduction

Language and music are highly developed and inter-related communicative systems employed and enjoyed by all known cultures. Similarities between the two are striking, suggesting that they may in fact share a common precursor (Wallin et al., 2000; Mithen, 2005). Linguists have long recognized that speech contains “music-like" features such as the frequency sweeps commonly associated with musical melodies (Steele, 1775). Similarities between the domains range from high level organization, such as structure (Lerdahl and Jackendoff, 1985), to low level processing, such as neural markers of semantic meaning (Koelsch et al., 2004).

Although some of the similarities between music and speech are innate and related to shared underlying processes (Patel, 2003), others appear to be the result of enculturation and experience. For example, the rhythmic patterns used in British and French instrumental music bear the fingerprints of composers’ native languages (Patel and Daniele, 2003a), a finding later broadened (Huron and Ollen, 2003) to include other nationalities (however, they did not appear in a survey of vocal music, VanHandel, 2009). Nonetheless, links between the domains are well-established—musical training develops skills in both speech and music (such as hearing speech in noise, or auditory brainstem responses to pitch and timing) at cortical as well as sub-cortical levels (Kraus et al., 2009; Strait et al., 2009).

Exploring the relationship between speech and music is informative for musicians, psychologists, neuroscientists, and linguists alike. The implications of such research are wide-reaching and can even inform clinical work, such as efforts to help stroke victims struggling to grasp the emotional tenor of speech (Borod et al., 2002). From a theoretical perspective, the study of acoustic cues such as pitch and timing in a musical corpus provides a useful complement to parallel analyses of speech. As musical notation allows composers to explicitly specify pitch and timing in notated scores, it is possible to explore exemplars of utterances precisely capturing the intentions of the sequence’s generator (i.e., a composer in the case of music or speaker in the case of speech). The use of musical notation also affords exploration of shifts in cue-use across centuries, providing insight into longitudinal changes—explorations challenging for speech originating from eras predating recording technology. Admittedly, analyses done decades or even centuries after a musical works’ creation are undertaken with different musical perspectives; however, they can still lend insight into our modern day hearing of historically significant works. Consequently, a corpus analysis of pitch and timing in a rich set of polyphonic music provides a useful complement to existing corpus analyses of speech.

### Three Cues Conveying Emotion

#### Communication through Pitch Height

Pitch is a salient cue for the interpersonal communication of emotion. In speech, higher pitch height is commonly associated with happiness along with submissiveness and diminished threat (Apple et al., 1979; Ohala, 1983). Similarly, sadness in speech is associated with lower pitches (Scherer, 2003). In music, passages transposed one octave (i.e., 12 semi-tones) lower consistently “sound sadder” whereas passages transposed one octave higher “sound happier” (Hevner, 1937). Although octave transpositions represent large changes in pitch height, even small differences can lead to salient perceptual consequences. For example, music lowered by two semitones is rated as “less tense” and “more pleasant” than music raised by the same amount (Ilie and Thompson, 2006). Manipulations pitting pitch against rhythm suggest that pitch may be the stronger cue: ratings of isochronous versions of happy passages (preserving the melody while removing rhythmic information) sound happier than monotone versions (preserving the rhythm while removing pitch changes) of the same passage (Schellenberg et al., 2000).

#### Communication through Timing

Timing is a powerful cue for conveying emotion in both speech and music, with faster rates conveying happiness and slower rates sadness (Scherer, 1995; Banse and Scherer, 1996; Sobin and Alpert, 1999). Speakers are more personally affected (based on self-evaluation as well as measures of heart rate and blood pressure) when speaking about sad topics if speaking slowly as opposed to at a neutral or a fast speed (Siegman and Boyle, 1993). In music, passages sound “sadder” when altered to be slower than their normal tempo, and “happier” when altered to be faster (Dalla Bella et al., 2001). Timing is such a powerful cue that participants consistently rate faster versions of pieces as happier (and slower as sadder) independent of other cues such as modality (Gagnon and Peretz, 2003) and harmonic complexity (Webster and Weir, 2005). Findings regarding music production complement those related to perception, with children’s spontaneous singing tempos being faster when asked to perform in a manner that would make the experimenter “happy” than when asked to make them “sad” (Adachi and Trehub, 1998). Although the 4-year-old children recruited for that study had likely previously experienced music in a variety of forms, these results complement other findings that certain cues are culturally invariant – such that Western-acculturated listeners can recognize certain aspects of emotional communication when listening to North Indian raga (Balkwill and Thompson, 1999). The fact that such cues can be recognized across cultures is consistent with the idea that these associations stem from physiological consequences of arousal level (Huron, 2012).

#### Modality^1^: both a Cue and a Classifier

Although pitch and timing are used in both music and speech, one powerful cue specific to music is that of mode, with major generally conveying a sense of happiness and minor of sadness. This association has a long history—Hevner (1935) drew up a list of adjectives thought to be communicated through major and minor keys, finding positive adjectives such as “happy,” “joyous,” and “exhilarated” associated with major keys and negative adjectives such as “sad,” “pathetic,” and “melancholy” associated with minor. Results indicate that regardless of musical training, intelligence, or talent, subjects consistently classified major-key pieces into the “happy” and minor-key into the “sad” categories, respectively. Major melodies are consistently perceived as “more positive” than minor after enculturation within the Western style (Gerardi and Gerken, 1995). Moreover, major melodies tend to be rated as sadder when altered to conform to the minor mode, and happier when altered to conform to the major (Gagnon and Peretz, 2003). The long-standing relationship between mode and emotion (Hevner, 1935; Rigg, 1937) is so well-established that it has even been used to assess clinical deficits in music processing (Peretz et al., 1998). Recognized associations between major/minor chords have also been useful in situations requiring mood induction (Houston and Haddock, 2007), and even studies exploring vision’s influence on auditory judgments. For example, major thirds sound “sadder” when accompanied by the facial expressions used to sing minor thirds (and minor sound “happier” when presented with the facial expressions for major thirds) in a multi-modal integration task involving singing (Thompson et al., 2008).

Consequently, mode has previously been used as an *a priori* classifier when analyzing large musical corpora. For example, Huron (2008) compared the pitch heights of major and minor key themes (i.e., monophonic melodies), noting the major themes were 1.1 semi-tones higher than minor. Comparisons of major vs. minor key pieces have also been used in explorations of dynamic markings across 140 unique musical works (Ladinig and Huron, 2010) as well as dynamic markings taken from samples of 48 pieces by eight different composers (Turner and Huron, 2008). Comparisons of major vs. minor key repertoire for percussion literature revealed interactions between the acoustic affordances of percussion instruments and the nature of repertoire written for them. Compositions for the xylophone, with its higher pitch range and shorter durations (necessitating faster attack rates) are overwhelmingly written in major keys. This contrasts sharply with composers’ more equal distribution of modalities when writing for the marimba—an instrument affording a wider range of pitches and attack rates (Schutz et al., 2008). In a different approach, the spectra of major intervals (i.e., intervals that differentiate the major mode from the minor) within a corpus of melodies share similarities with the spectra of excited speech, and minor intervals share similarities with the spectra of subdued speech (Bowling et al., 2010).

Although numerous studies of cue use have documented parallels between music and speech, the degree of similarity is in some cases dependent upon the musical era in question. For example, Post and Huron (2009) showed that major key variations-on-a-theme exhibited faster tempo markings than minor within the Baroque, Classical, and Modern eras. However, composers in the Romantic era broke with this pattern, which is to some extent indicative of broad changes in cue use within this musical period (Horn and Huron, 2015). As such these findings admittedly pose challenges for deriving universal rules of musical communication – an issue we will revisit in the discussion. As with all cues, mode influences but does not dictate a passage’s emotional tenor. It is possible to write minor key melodies that do not sound sad by using high pitch heights and fast timings (e.g., the theme from Mozart’s Symphony No. 40 in g minor). Similarly, it is possible to write major key melodies that are less than joyful. Therefore, the use of modality as an *a priori* classifier may admittedly risk oversimplifying a complex issue. Nonetheless, exceptions to general tendencies do not invalidate their predictive value in other contexts. It is possible that factors such as parallels with speech are weighted less heavily in certain eras, and/or that undermining expectations can be artistically desirable. For example, jazz’s violation of certain musical norms is part of its appeal, and does not mean the “rules” it is circumventing fail to describe normative musical practices. Consequently, formally assessing whether major key pieces exhibit cues corresponding to happy speech (and minor to sad) is one way of empirically testing the correlation of cues previously documented as playing significant roles in order to better understand the complex relationship between low level cues, modality, and emotion.

### Two Approaches to the Study of Cue Use

Many previous studies of cue use can be grouped into two broad methodological approaches. The *perceptual consequences* approach consists of exploring the perceptual effects of specific low-level cues manipulated *a priori* (i.e., through changes in pitch height or timing information), allowing for analysis of how these manipulations affect subsequent evaluations. Many of these studies in the realm of speech investigate decoding reliability between listeners (Apple et al., 1979; Banse and Scherer, 1996; Simon-Thomas et al., 2009), although this approach has also been used to measure the correlation between acoustic cues and physiological reactions such as blood pressure and heart rate (Siegman and Boyle, 1993). Perceptual consequences approaches in music have been useful in exploring the strength of one cue relative to another, such as timing vs. mode (Gagnon and Peretz, 2003) or rhythm vs. melody (Schellenberg et al., 2000).

Alternatively, the *corpus analysis* approach analyzes exemplars of speech or music known to convey certain emotions in order to determine the cues responsible for this communication. For example, Banse and Scherer (1996) analyzed the mean pitch, speech rate, and intensity of speech utterances to examine their relationship to an excerpts’ emotional tenor. Similarly, other speech research explores the role of individual cues in non-lexical communication, including the use of fundamental frequency (Ohala, 1984), and pitch and volume levels and variance, speech rate, and pauses (Sobin and Alpert, 1999).

Although both the perceptual consequences and corpus analysis approaches offer valuable insight, to the best of our knowledge they have not been equally employed in speech and music research due in no small part to corpora asymmetry. Given the ubiquity of speaking ability, assembling a corpus of speech tokens expressed in a variety of emotional tenors (Banse and Scherer, 1996), or of unrehearsed speech produced in known emotional states (Siegman and Boyle, 1993; Bachorowski, 1999) is a time consuming yet manageable endeavor. However, although humans are well-practiced at generating sentences utilizing emotional cues, far fewer are proficient at generating melodies. Given this disparity between the ubiquity of “effective speakers” and the relative paucity of “effective composers,” assembling a parallel musical corpus is problematic. It is obviously impossible to ask historically significant composers to create balanced sets of happy and sad pieces. We do note, however, one study asking five contemporary composers to create novel melodies specifying emotions such as “joy,” “sorrow,” “peacefulness” etc. Naïve participants then evaluated the stimuli, and were generally adept at decoding the intended emotions (Thompson and Robitaille, 1992). This technique of generating “controlled” melodies supplements more traditional perceptual consequences research manipulating targeted aspects of pre-composed melodies. Here, we complement both of those approaches by exploring polyphonic excerpts of historically significant works created by widely admired composers in a naturally balanced corpus of major and minor key pieces.

Although assembling appropriate corpora can be difficult, previous corpus analyses of music illustrate their value when suitable repertoire can be identified. These studies offer glimpses into a wide arrange of patterns, however they generally focus on relatively simple musical qualities: the relationship between isolated dynamic (Turner and Huron, 2008; Ladinig and Huron, 2010) and/or tempo markings (Post and Huron, 2009) and modality, or rhythmic variations in monophonic melodies (Huron and Ollen, 2003; Patel and Daniele, 2003a,b). Some do explore more complex parameters, such as the relationship between pitch height and note duration, as well as the relationship between rate/speed and tessitura or pitch range (Broze and Huron, 2013). However, to the best of our knowledge there has not been a corpus analysis exploring pitch height of all voices in polyphonic music (let alone in conjunction with timing), despite the widely recognized importance of these cues in natural speech (Ohala, 1984; Banse and Scherer, 1996; Sobin and Alpert, 1999; Johnstone and Scherer, 2000).

### Approach and Goals for this Study

Our analysis is based on excerpts containing complete musical textures (accounting for all voices/harmonies/etc. rather than melody alone) of music that has become a staple of the Western canon. By engaging in a traditional score-based analysis we aimed to capture important musical nuances that shape the listening experience. Our goal was to inform discussion surrounding the extent (and limits) of parallels in the communication of emotion between speech and music, providing practical insights for composers/arrangers, as well as theoretical insights regarding non-verbal communication. Previous research demonstrates that manipulations to melodies to make them major (Gagnon and Peretz, 2003), higher in pitch (Hevner, 1937; Ilie and Thompson, 2011), and/or faster (Dalla Bella et al., 2001) all result in happier sounding passages (and conversely minor keys, lower pitches, and slower timings lead to sadder sounding passages). Here, we explore the degree to which unaltered polyphonic music by great composers “naturally” uses these cues, complementing previous studies using manipulations of single line melodies or generation of novel monophonic melodies used exclusively for experimental purposes.

#### Choosing a Corpus

One challenge for this type of investigation is choosing a suitable corpus “balanced” with respect to modality. This is crucial given modality’s well-recognized importance in communicating emotion (Hevner, 1935; Rigg, 1937; Peretz et al., 1998; Schellenberg et al., 2000; Gagnon and Peretz, 2003; Hunter et al., 2010), which has necessitated careful consideration in other corpus based work (Turner and Huron, 2008; Ladinig and Huron, 2010). Balancing modality becomes particularly challenging when attempting to assemble a corpus of historically significant music frequently performed and heard. Although symphony themes might initially seem promising, their distribution generally tilts heavily toward major. For example only ten of Haydn’s 100+ symphonies are in minor keys. Similarly only two of Mozart’s 40+ symphonies^2^ are written in minor (Tan et al., 2010, p. 251). Beethoven’s output is only slightly more balanced with two of nine symphonies written in minor keys. Later composers are no more helpful with respect to key balancing, as the total number of symphonic compositions generally decreased post-Beethoven, with a shift to writing fewer works longer in duration (Larue et al., 2015). The bias toward/preference for major keys is not confined to classical music – over 80% of a 1186 piece jazz corpus assembled for use by Broze and Shanahan (2013) are in major keys (Shanahan, personal communication July 9, 2015). Additionally, a preference for major over minor was noted in a corpus of rock music derived from *Rolling Stone* magazine’s list of the “500 Greatest Songs of All Time” (Temperley and de Clercq, 2013).

In the current analysis, we settled upon 24-piece sets of keyboard miniatures as a useful way of exploring the compositional choices of renowned composers within the context of a “balanced” corpus. These sets are well-suited for comparative analyses as they provide a corpus not only balanced with respect to modality (i.e., 12 major and 12 minor key pieces), but also with respect to tonal areas (the “pitch home” for each piece). Written by a single composer within a relatively short period of time, such compositions provide a targeted “snapshot” of one musician’s approaches, which offers certain advantages over a corpus drawing upon many disparate composers. The use of sets from different time periods affords the opportunity to explore changes in cue use across musical eras.

#### Merits of Focusing on Keyboard Literature

Although composers have written 24-piece sets for a variety of instruments, literature for the keyboard holds several advantages. First, the piano (and its precursors) is less capable of varying the timbre of individual notes (relative to other instruments), yet possesses a large pitch range and great flexibility/nuance in its control of timing. Keyboard music thus affords clear quantification of the cues of interest (pitch and timing). Secondly, the modern piano and its historical precursors were the instrument of choice for several centuries, resulting in a sizable repertoire including multiple 24-piece sets (Beuerman, 2003). We chose to focus on works by Bach and Chopin as these are arguably among the most widely played pieces of this nature. For example, pieces from these sets are featured in the Royal Conservatory of Music standards lists at multiple competition levels (Royal Conservatory of Music, 2015).

Based on previous findings that happy speech exhibits a higher fundamental frequency and a faster rate than sad speech, we hypothesized *a priori* that major-key (nominally “happy”) pieces in our corpus would be on average higher in pitch and faster in timing than minor-key (nominally “sad”) pieces. After completing our analysis we noticed several additional patterns, which we report as *post hoc* observations. Our goal is to complement previous work involving manipulations of musical stimuli illustrating that such cues *can* be effective (Hevner, 1937; Schellenberg et al., 2000; Gagnon and Peretz, 2003) by exploring *how* they have been used through a rigorous, note-by-note analysis of 24 piece sets widely studied, performed, and enjoyed by students, musicians, and audiences alike. Our analysis is based on complete excerpts (i.e., accounting for all voices/harmonies with our range of interest, rather than only melodies) by two widely renowned composers, and will inform our understanding of the extent (and limits) of parallels in the communication of emotion between speech and music. Consequently, it will provide insight useful for composers, arrangers, and songwriters interested in understanding the mechanics of emotional communication. Additionally, it will inform efforts by psychologists, neuroscientists, and linguists to understand interpersonal communication in speech.

## Materials and Methods

### Repertoire

Our corpus consisted of three 24 piece sets; with each set containing one piece in each of the 12 major and minor keys. These sets are frequently performed in a wide range of musical competitions, recitals, and juries, giving this study broad relevance to common musical activities. The selection included (1) Preludes and (2) Fugues from the first book of Johann Sebastian Bach’s *Well-Tempered Clavier*. For stylistic contrast we also included (3) Frédéric Chopin’s *Preludes (Op. 28)*^3^. These three corpora yielded 72 individual compositions. We used the Kalmus Classic edition of the *Well-Tempered Clavier* (Kalmus number 03036) edited by Hans Bischoff (Bach, 1883), which contained tempo markings by the editor for each piece. Although the lack of explicit tempo markings during the Baroque era makes it impossible to determine Bach’s precise intentions, for our purposes we merely required reasonable tempos chosen by a musician with stylistic knowledgeable naïve to our hypotheses. In our “Discussion” section we provide details on a separate analysis comparing the results of using tempi from seven different editions, making use of a survey by musicologist Willard Palmer (Bach, 2004). That analysis demonstrates that our outcomes would not have differed meaningfully had we used any of the seven editions (that analysis appears later in our Discussion as we learned of his survey after completing cue extraction). For analysis of the Chopin, we used the Breitkopf and Härtel (Chopin, 1839) edition of the *Preludes.* As with most editions, this included general tempo descriptions (i.e., “Agitato”) rather than precise metronomic markings. We therefore used the metronome values (i.e., quarter = 72) provided by Willard Palmer in a table summarizing Kohler’s recommendations (Chopin, 1992)^4^ to calculate the attack rate. To the best of our knowledge this is one of the few explicit tempo guidelines for the Chopin, and provides a useful reference from a respected editor unfamiliar with our hypotheses.

In order to minimize the amount of time excerpts spent in other keys after modulation (a musically common occurrence), we confined our analysis *a priori* to the first eight complete measures of each piece. We considered measures complete if at least half of their total number of beats contained notes (i.e., a four beat measure must have at least two beats with notes as opposed to rests). We set this criterion to avoid an undue weighting of pick-up notes/partial measures (these omitted measures were distributed evenly, with three in major and three in minor keys)^5^.

### Pitch Height Analysis

We operationalized pitch height so as to find the “center of gravity” of pitch within each measure through the following process. We assigned each note a numeric value corresponding to its location on a standard 88-key keyboard (A0 = 1, C8 = 88; the letter refers to the pitch-class, while the number refers to the octave). We also calculated a weighting for each note based on relative duration, ensuring longer notes were not overpowered by series of short notes (i.e., a half note had the same weight as four cummulative eighth notes). Quarter notes, for example, received a value of 1, half notes a value of 2, and eighth notes a value of 0.5^6^. For the purposes of the pitch analysis, notes tied within a measure were assigned a single duration equal to their sum, but those tied across the bar line were broken up and assessed separately (each with their own duration value) so that their contribution was reflected within each measure. This approach offered the ability to extract precise pitch values on a measure-by-measure basis, rather than giving only summary values for an entire passage. Segmentation by measure avoids under-weighting the pitch height of measures with fewer vertical notes (i.e., a single whole note sounding for 2 s in one measure rings for the same amount of time as a four-note chord sounding for 2 s in the next).

We chose to calculate pitch by measure, as it is the smallest common unit across all 72 pieces feasible for both pitch and timing cues. Beat-by-beat analysis would be problematic as many pieces differ in the number of beats per measure. For example Bach’s *d minor Fugue* (No 6) has three beats per measure, his *B Major Fugue* (No 23) has four, and his *f# minor Fugue* (No 14) has six beats per measure. Similarly, it would not be possible to perform a comparative analysis on a note-by-note basis as pieces in different time signatures do not align vertically. For example, the first measure of Bach’s *Prelude in c minor* (No. 1) in 4/4 time contains four sixteenth notes per beat, whereas the first measure of his *Prelude in E Major* (No. 9) in 12/8 contains three eighth notes per beat (only the first note of each grouping would align vertically). Similarly, the complex rhythmic structure of Chopin’s *Prelude in f# minor* (No. 8) featuring running 32nd notes in the right hand and sextuplet groupings in the left has few points of temporal overlap with Chopin’s *Prelude in E Major* (No. 9) featuring eighth note triplets.

We accounted for ornaments such as trills (i.e., rapid alternations between two notes) by using the average value of the notes in the ornament [e.g., a trill between a C4 (40) and C#4 (41) would receive a value of 40.5], and assigning written-out grace notes a duration weighting equal to that of a 32nd note (0.125). For the Chopin *Preludes*, this duration value was then subtracted from the previous note, while in the *Well-Tempered Clavier* this duration was subtracted from the subsequent note. We used this approach in order to be consistent with performance practice—grace notes in Romantic music are performed prior to the beat, whereas in Baroque music they are (almost always) played on the beat^7^.

On occasion we encountered notes with multiple stems. This occurred primarily when voices in the Bach *Fugues* crossed, and in those instances we used the note weight corresponding to the longer duration^8^. We also observed multiple stems in different contexts in the Preludes – such as Chopin’s *Prelude No. 1*. After listening repeatedly (MP was a piano student at the time), we chose to use the shorter duration of these notes as they generally occurred within the context of rapid passages, and consequently function more according to the shorter, rather than the longer values. For the sake of consistency, we chose to use the shorter durations for all of Bach’s and Chopin’s *Preludes*. These exceptional cases occurred within only 47 (22 Bach and 25 Chopin) out of the 576 measures analyzed, and our decisions affected major and minor key pieces similarly. Moreover, it is important to note that the number of measures with double-stemmed notes did not meaningfully favor one mode in particular – 7% of measures in major-key preludes featured double-stemmed notes, compared with 10% of measures in the minor-key preludes.

**FIGURE 1:**
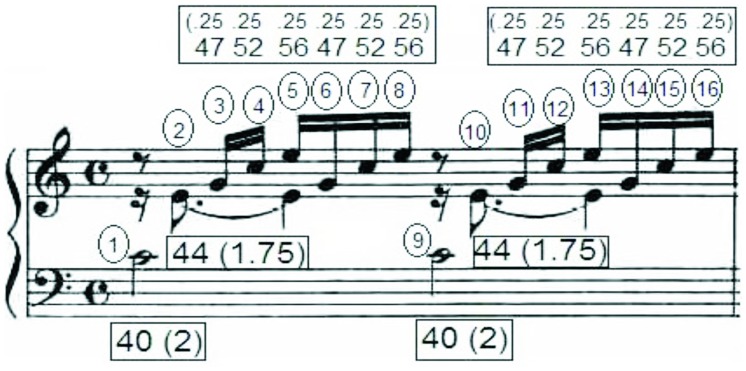
**Sample analysis for first measure of *Prelude no. 1 in C major* by J. S. Bach.** Pitch height analysis is indicated in the boxes, with each note receiving a pitch number corresponding to the standard 88-key piano keyboard, and a relative duration value centered around quarter note = 1 (in parentheses). Each of the 16 attacks is enumerated in circles. Therefore, the total number of attacks per second for this measure is 7.467 (using a metronome marking of 112 beats-per-minute), and its average pitch height is 44.67.

We calculated the average pitch height (i.e., “center of gravity”) for each measure by summing the weighted values for that measure and dividing by that measure’s total sum of durations. As shown in **Figure 1**, in the first measure of Bach’s *Prelude in C Major*, the total weighted values = 469, the total sum of durations = 10.5, yielding a weighted average of 469/10.5 = 44.67. We then calculated an average pitch height for the first eight measures of each of the 72 compositions in our corpus (12 major and 12 minor pieces within each of three sets), resulting in 576 values for pitch height.

### Attack Rate Analysis

Explorations of timing in music are frequently based on tempo – the number of a beats per minute. Although using tempo as an index of timing has certain advantages (simplicity chief among them), it is somewhat inexact for paralleling the way timing is measured in speech, which is generally discussed in terms of articulation rate (Johnstone and Scherer, 2000; Scherer, 2003). As such, we operationalized our assessment of timing by using *attack rate*^9^ (i.e., the number of note attacks per-second). Attack rate holds three advantages: (1) it is more sensitive to anomalous situations in which pieces at fast tempos may in fact contain a slow rate of attack through the use of long notes, (2) it captures measure-to-measure changes in timing information within passages written at a stable tempo, and (3) it offers a closer parallel of speech researchers’ use of articulation rate (i.e., number of speech articulations per second).

Because attack rate reflects both rhythmic structure and tempo, determining reasonable tempi was still important for our study. Although by definition no tempo can be considered the *only* appropriate choice, suggestions given by a respected editor represent *one* reasonable interpretation. More importantly for our purposes, they provide values from expert musicians naïve to our hypotheses (we later include a formal analysis demonstrating that our findings are stable across seven different editorial sets of tempi). Our focus on scores rather than recordings is consistent with previous efforts by empirical musicologists to analyze timing in music composed prior to the widespread use of recording technology (Turner and Huron, 2008).

We summed the number of attacks within a single measure (counting chords as a single attack), and divided the attacks by the number of that measure’s beats. We then calculated attacks-per-second by dividing the metronome values (in beats per minute) by 60, and multiplying this value by the number of attacks per beat. For instance, the example in **Figure 1** has 16 attacks over four beats, giving an average of four attacks per beat. The metronome marking was 112 beats per minute, resulting in 7.47 attacks per second. We applied this method to the first eight measures of all 72 compositions within our corpus (12 major and 12 minor pieces within each of three sets), resulting in 576 timing values.

**FIGURE 2:**
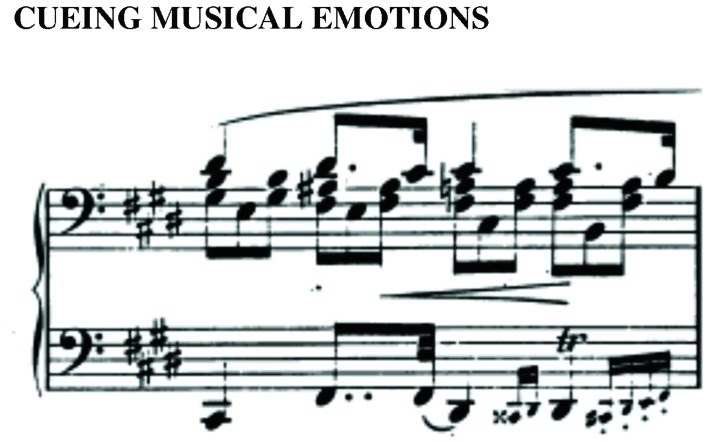
**Chopin *Prelude no. 9 in E major*, measure 4.** Because the exact alignment of multiple grace notes (e.g., in beat 4) with the right hand would be ambiguous, we elected to count each grace note and each primary note as an attack.

We did not count ornaments separately on the grounds that they function as “decorations” of a note, and each attack is not explicitly notated individually in a score. However, we did count grace notes provided they were notated individually. Because of the ambiguity of grace notes in lining up with another note (e.g., in **Figure 2**, the exact alignment of the four grace notes in the left hand with the attacks in the right hand are impossible to determine), we chose simply to give each grace note one attack^10^. Crucially, we used the same practice across all major and minor key pieces. As with previous notational challenges, given the infrequent and balanced occurrence of grace notes (4% of measures in major-key preludes and 3% in minor-key preludes), it is unlikely that different decisions regarding the treatment of pickup beats, grace notes, double stemming, or other nuanced notational issues would lead to qualitatively different results. For the sake of transparency and to aid other researchers interested in future extensions and/or replications, Table A1 contains a full list of the challenging issues encountered throughout the analysis broken down by corpora.

## Results

**FIGURE 3:**
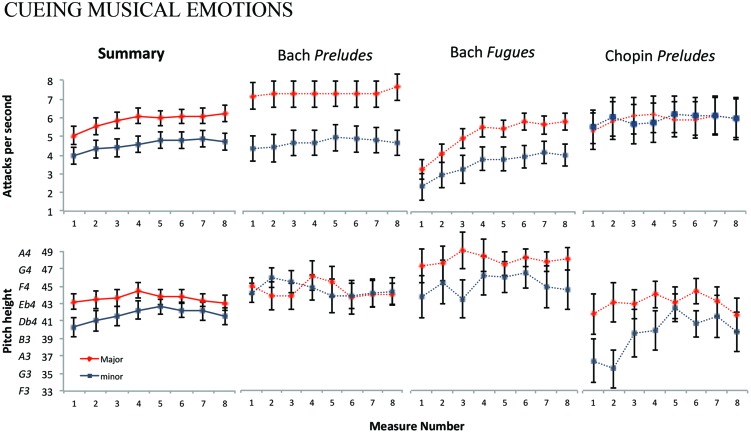
**The two panels on the left show a summary of average pitch height and attack rate per measure for all 72 pieces, with the major-key pieces represented by a solid blue line and the minor-key pieces represented with a solid red line.** The other six panels show average pitch height and attack rate per measure for the 24 preludes from the *Well-Tempered Clavier*, 24 fugues from the *Well-Tempered Clavier* and the 24 preludes from Chopin’s *Preludes*, each drawn with dashed lines. Error bars indicate 1-standard error about the mean.

Our most important finding relates to our two *a priori* hypotheses: the 36 major-key (*M* = 43.6, *SD* = 5.07) pieces were roughly a major second (i.e., two semi-tones) higher in pitch than the 36 minor (*M* = 41.8, *SD* = 6.09) – approximately E4 relative to D4 on the piano. Additionally the major pieces (*M* = 5.86, *SD* = 2.52) were 28.5% faster than minor (*M* = 4.56, *SD* = 2.71). Both of these outcomes were consistent with the use of these cues in happy vs. sad speech. These results are summarized in detail in **Figure 3.** As our eight values (i.e., eight measures) for pitch height and attack rate per piece were not independent, we employed a linear regression model using ordinary least squares,^11^ with corpus (Chopin’s *Preludes*, Bach’s *Preludes*, Bach’s *Fugues*), mode (Major, minor), and measure number (1–8) as factors. We set an α level of 0.05 to assess significance throughout. The results are reported from the analysis of variance (ANOVA) table for the fitted model, which estimates the significance of omnibus *F*-tests for multi-level factors and, where applicable, their interactions with other predictors.

### Full Corpus

With respect to attack rate, mode was a significant predictor across the three corpora (*F*_6,564_ = 10.91, *p* < 0.001). The effect of corpus itself was also significant (*F*_8,564_ = 10.89, *p* < 0.001), as well as its two-way interaction with mode (*F*_6,564_ = 6.18, *p* < 0.001) and its two-way interaction with measure number (*F*_6,564_ = 2.30, *p* = 0.0332). No other interactions were statistically significant. With respect to pitch height, mode was also a significant predictor across the three corpora (*F*_6,564_ = 5.15, *p* < 0.001). Corpus was also significant (*F*_8,564_ = 11.53, *p* < 0.001), as well as its two-way interaction with mode (*F*_4,564_ = 3.14, *p* = 0.0143). However, in contrast to its relationship to attack rate, measure number was not a significant predictor of pitch height. No other interactions were statistically significant.

Having tested our two *a priori* hypotheses on the corpus as a whole and having found corpora to be a significant predictor, we then analyzed each corpus separately to gain a more fine-grained view. This exploration offers additional insight into the use of pitch and timing within each set, affording subsequent discussion of how within-set differences (i.e., pitch height in major key Bach *Fugues* vs. minor key Bach *Fugues*) vary from set to set (i.e., comparing differences in Bach’s *Preludes* to differences in Bach’s *Fugues*).

### Individual Corpora (Attack Rate)

With respect to attack rate, mode was a significant predictor for the Bach *Preludes* (*F*_2,188_ = 33.31, *p* < 0.001) with major-key pieces (*M* = 6.87, *SD* = 2.06) approximately 55% faster than minor (*M* = 4.42, *SD* = 2.08). It was also significant for the Bach *Fugues* (*F*_2,188_ = 16.00, *p* < 0.001) where the major key pieces (*M* = 4.86, *SD* = 1.72) were 43% faster than minor (*M* = 3.41, *SD* = 2.04). Yet it was not a significant predictor for the Chopin *Preludes*, which exhibited similar values for major (*M* = 5.86, *SD* = 3.15) and minor (*M* = 5.87, *SD* = 3.26) pieces. Measure number was a significant predictor only for the Bach *Fugues* (*F*_2,188_ = 11.89, *p* < 0.001), likely reflecting the characteristic addition of new voices measure by measure. We found no significant interaction between measure number and mode within any of the three corpora.

### Individual Corpora (Pitch Height)

With respect to pitch height, mode was not a significant predictor within the Bach *Preludes*, with a negligible difference between major [*M* = 43.0 (∼Eb4), *SD* = 4.40] and minor [*M = * 43.2 (∼Eb4), *SD* = 4.03]. In the Bach *Fugues*, major pieces [*M* = 46.0 (F#4), *SD* = 4.52] were 2.5 semitones higher than minor [*M* = 43.5 (∼E4) *SD* = 6.18], making it a significant predictor (*F*_2,188_ = 5.08, *p* = 0.007). In the Chopin *Preludes*, major key pieces [*M* = 41.7 (∼D4), *SD* = 5.37] were 3.2 semi-tones (just over a minor third) higher than minor [*M* = 38.6 (∼B3), *SD* = 6.51], making mode a significant predictor of pitch height (*F*_2,188_ = 7.88, *p* < 0.001). Measure number was not a significant predictor of pitch height in any of the three corpora, nor was its interaction with mode.

## Discussion

Our goal was to assess the hypothesis that pitch and timing cues were used in a manner consistent with previous perceptual consequences research on music within a corpus of historically significant and widely performed music. Using our operationalized definitions of pitch height and timing, we found that major key pieces were in fact 29% faster in attack rate than minor (however, we note that this difference was driven entirely by Bach). Additionally, major key pieces in this corpus were approximately a major second (i.e., two half-steps) higher – a distance previously shown sufficient to evoke changes in emotional tenor (Ilie and Thompson, 2006). Both of these results were consistent with our initial hypotheses. Although parallels between the communication of emotion in speech and music have been well-documented using perceptual consequences approaches manipulating melodies, we believe this to be the first corpus analysis study documenting such a relationship in polyphonic excerpts of widely performed music (although we note previous exploration of isolated dynamic markings by Turner and Huron, 2008; Ladinig and Huron, 2010 and of single line melodies lines by Huron, 2008). Consequently our findings complement existing research on music-language parallels by providing converging evidence derived from a “natural” musical setting.

It is possible that some of the pitch difference between major and minor pieces resulted from structural differences between major and minor keys. Namely, the lowered third, sixth, and seventh in natural minor (vs. major) scales results in a slightly lower average pitch height for minor vs. major instantiations of a melody. We note one previous analysis of pitch height in major and minor key themes calculates that the structural differences in minor scales results in a small lowering of average pitch height – conservatively approximated as 3/7 of a semitone as a consequence of lowering three scale degrees by a half step (Huron, 2008). Huron concluded this structural effect was insufficient to explain the 1.1 semi-tone difference in pitch height observed between major vs. minor key themes within his corpus. As our pitch difference was larger than 1.1 semi-tones (it included a balanced sample of major/minor keys and harmonic as well as melodic information), it is unlikely that it would explain our finding of a two semi-tone difference. Nonetheless we re-ran our pitch height analysis lowering all major key pieces by 3/7 of semitone, finding that mode remained a significant predictor across the three corpora (*F*_6,564_ = 3.96, *p* < 0.001). Consequently this structural distinction cannot fully explain our observed difference in pitch height.

It is worthwhile to note that our data consists entirely of music written by widely renowned composers that is considered common repertoire amongst pianists, rather than tone sequences or melodies generated for a particular experiment, melodies chosen for their novelty, or manipulations of pre-composed music. As our corpus consisted of particular sets of music by Bach and Chopin, future research is needed to explore whether these findings generalize more broadly. However, these results provide a novel demonstration that a corpus of historically significant music uses these cues in a manner consistent with that documented in experimental contexts – whether or not this choice was deliberate.

### Comparing Corpora

Although analysis of the corpus as a whole revealed cue use consistent with speech, the specific major-minor differences found within each of the three corpora varied considerably. As we did not make *a priori* predictions about inter-corpus differences, we consider the following tests *post hoc* observations included for the sake of thoroughness. Note that in the following discussion of inter-corpus differences we do not directly compare cues between corpora (i.e., pitch height in Bach’s major key *Fugues* in relation pitch height in Chopin’s major key *Preludes*). Rather, instead we compare differences in the use of cues in major vs. minor pieces within a single corpus, contrasting this within-corpus difference across sets. In other words, we explore how the distinction in pitch height between major and minor pieces in Bach’s *Fugues* differed from the distinction in pitch height between major and minor pieces in Chopin’s *Preludes*, etc.

Bach’s *Preludes* exhibited the greatest attack rate distinction, with the major key pieces 55% faster than minor. Bach *Fugues* featured a smaller difference of 43% between major and minor, and Chopin’s *Preludes* exhibited none. Interestingly, this within-corpora pattern of attack rate distinction was reversed with respect to pitch height. Bach’s *Preludes* exhibited no significant distinction in pitch height between major and minor key pieces, whereas Bach’s *Fugues* exhibited a 2.5 semitone difference and Chopin’s *Preludes* 3.1 semitones (slightly larger than a minor third). Consequently these three corpora appear to “trade-off” with respect to within-corpus cue differentiations by modality. Although future explorations designed to formally assess this issue are needed to draw strong conclusions, we found this pattern intriguing as it complements previous observations that trade-offs in pitch and timing may “make the experience richer and more interesting” and that composers may intentionally attempt to balance these cues “even if this knowledge is implicit rather than explicit” (Hunter et al., 2008).

### Historical Changes in the Use of Mode

Our initial hypothesis with respect to timing was that major key pieces would be faster than minor. Although this prediction was borne out for the corpus as a whole, the lack of difference in Chopin demonstrates that this “main effect” of modality was not uniform. The degree to which this poses challenges for the larger theoretical framework with respect to parallels between speech and music is to some extent an open question requiring future research. However, in contemplating these differences, it is helpful to consider the different musical eras in which the two composers worked. In contrast to the lack of difference in attack rate between major/minor pieces in this corpus by Chopin (1810–1849), major key pieces within this corpus by Bach (1685–1750) exhibit a strong difference in attack rate – 49% when averaged across the two sets.

Although we are unable to draw firm conclusions about changes in cue use over time from this sample of two composers, we do note that this finding is consistent with other work showing a shift in cue use during the Romantic era (the period of musical history in which Chopin’s music falls). For example, in comparing tempo markings in 331 compositions across three musical time periods (Baroque, Classical, and Romantic), minor key pieces were slower than major in the Baroque and Classical eras, but faster in the Romantic (Post and Huron, 2009). Similarly, an analysis of the dynamic markings taken from six different 24-piece preludes sets (144 pieces in total, with 72 major and 72 minor) found evidence of differences in the use of dynamics in the Romantic era (relative to its precursors) after separately accounting for early and late romantic-era compositions (Ladinig and Huron, 2010). An exploration of 8117 excerpts composed between 1700 and 1950 found that use of the minor mode itself increased dramatically in the Romantic era along with a general shift in the use of cues (Horn and Huron, 2015).

It could be argued that our finding of differences in the use of timing between Bach and Chopin along with previously noted changes in the use of modality within the Romantic era poses an interesting challenge to generalizations regarding cue use, affect, and modality. However, the fact that certain trends are exhibited in most but not all musical eras does not render them irrelevant – it simply means they should be interpreted carefully. Although musical decisions made by composers undoubtedly reflect large historical forces such as our species’ evolutionary history (and hence physiologically based cue associations), this history holds a vote rather than a veto. Cultural pressures, artistic decisions, and even intentional violations of expectations all play important roles. Cue use can shift considerably even within a generation. For example, popular music within the last 40 years has exhibited clear changes in the relative prevalence of minor key pieces (Schellenberg and von Scheve, 2012). Consequently we see this documentation of differences in the use of timing in major vs. minor pieces in the Baroque and Romantic eras as contributing to broader empirical exploration of music’s historical evolution. Additionally, it provides another piece of insight into the complex relationship between low-level musical cues, high-level structural considerations, and audience responses. Future research exploring changes in cue use over musical history will help to clarify complex multi-faceted issues at the intersection of cultural, physiological, and artistic pressures.

### Properly Assessing Tempo

Although our primary interest was in differences between major and minor attack rates (rather than absolute values of attack rates for particular pieces), our analysis by definition required establishing reference tempos. We chose to use editorial tempo markings to side-step ambiguities with respect to performer interpretation—an issue that often goes unnoticed when using single performances to represent a composition’s “canonical” realization. However, we recognize two potential objections to this approach: (1) these tempo markings came from later editors rather than the composers themselves, as explicit tempo markings were not common in their time, and (2) the “correct” tempos are in some cases subject to debate, with disagreement amongst respected editors. As we were able to find precise notated tempo guidelines for Chopin from Willard Palmer’s table referencing Koheler’s tempi (Chopin, 1992), we consider this to be primarily an issue with respect to the Bach.

After completing our initial analysis of Bach’s pieces using metronome markings supplied by Hans Bischoff, we learned musicologist Willard Palmer had compiled a list of tempo recommendations from two commentators (Keller, Bodky) and five editors (Czerny, Bischoff, Mugellini, Hughes, and Bartok) – which he summarized in a table within his edition (Bach, 2004). As would be expected, tempo recommendations did not always agree for individual pieces. For example, Keller tended to recommend slower tempi for Bach’s fugues than did Bischoff (the editor whose markings formed the basis of our timing calculations). However, our assessment relied not on the absolute attack rates, but rather *relative differences* in attack rate between major and minor key pieces. Therefore the critical question for our purposes is whether the tempos recommended by different editors would lead to different relative outcomes. If one editor consistently recommended faster tempos, differences between major and minor key attack rates would not be affected. Similarly, an editor’s selection of relatively faster tempi in some pieces and relatively slower tempi in others (i.e., a wider “spread”) would not necessarily affect the major/minor comparison of interest.

**FIGURE 4:**
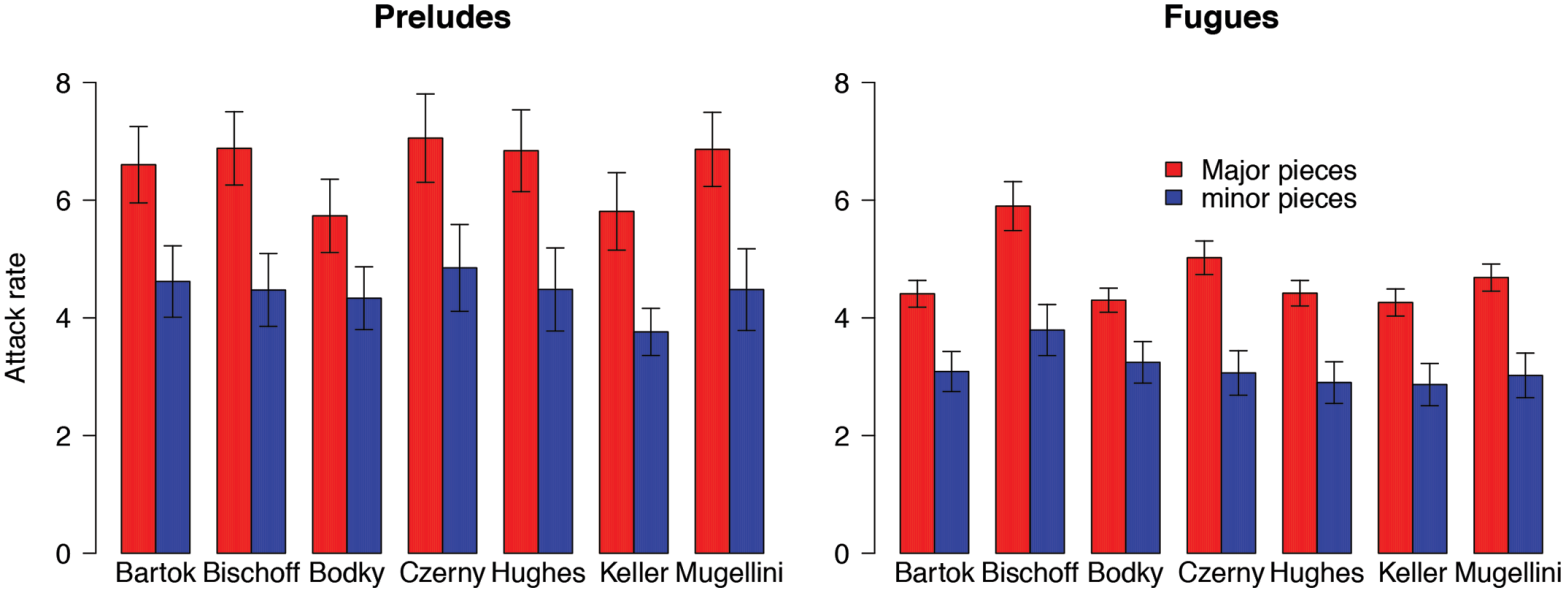
**Attack rates for major and minor key pieces by Hans Bischoff (as calculated in our analysis), as well as the four additional editors (Bartok, Czerny, Hughes, Mugellini) and two additional commentators (Bodky, Keller) discussed by musicologist Willard Palmer.** Results are plotted separately for *Preludes* **(left)** and *Fugues* **(right)**. Note that major key pieces were faster than minor for both *Preludes* and *Fugues* in all seven editions. Error bars indicate 1-standard error about the mean. For additional information (including visualizations) regarding the specific tempi recommended by each editor/commentator, please visit: http://maplelab.net/bachTempi

To explore this issue, we re-calculated the attack rates resulting from the tempi outlined by both commentators and four additional editors^12^. Our analysis revealed that that despite diversity with respect to tempo recommendations of individual pieces, the attack rates of major key pieces were faster than those of minor *for all seven interpretations* (**Figure 4**). A formal assessment using our previously described approaches demonstrated that although mode was a significant predictor of attack rate (*F*_7,322_ = 9.98, *p* < 0.001), the particular editor/commentator was not (*F*_12,322_ = 0.59, *p* = 0.846). Crucially, there was no significant interaction between mode and editor/commentator (*F*_6,322_ = 0.29, *p* = 0.941). Consequently we conclude that attack rate for major key pieces is faster than minor in both Bach’s *Preludes* and his *Fugues* – regardless of which editors’ tempi are used.

### Other Observations

Our results hold several additional points of interest that might be of use to other scholars. First the overall difference in attack rates between major and minor keys was larger relative to the difference in pitch height. This is consistent with findings in perceptual (Gagnon and Peretz, 2003) and developmental studies (Dalla Bella et al., 2001) on the importance of timing in conveying emotional information. Second, the average pitch height over the eight measures forms an “arch shape,” with the middle measures higher in pitch height than the first and last. Such arches are ubiquitous not only across musical styles (Huron, 1996, 2006) but even in non-human animal communications such as bird song, a finding thought to arise from physiological constraints (Tierney et al., 2011). Third, Bach’s *Fugues* feature an increase in attack rate over the eight measures, and especially within the first three, reflecting increased rhythmic activity as voices enter and the texture thickens. Finally, we note that Bach’s *Well-Tempered Clavier* is both narrower in range and higher in overall pitch than the Chopin *Preludes* (**Figure 3**). This may be a reflection of the extended range of the piano in Chopin’s time (Ripin et al., n.d.).

### Future Research

There are several potential avenues for future exploration. The first lies in looking at the communication of basic emotions other than happiness and sadness (such as anger or fear), or dimensional models representing valence, energy, and tension (Russell, 1980; Ilie and Thompson, 2006). While selected here for their clear distinction (both perceptually and in their cue use), future research might explore emotions more ambiguous with respect to valence. For example Terwogt and van Grinsven (1991) noted that some adult participants responded to Tchaikovsky’s “Swan Lake” as positive, with the reason that the piece felt “calm,” “composed,” or “serene.” Terwogt and van Grinsven also noted the difficulty in distinguishing between two negative emotions: anger and fear, both of which would share similar acoustic cues. Obtaining perceptual ratings of the excerpts analyzed would be helpful in pursuing this issue.

A second area of future work lies in possible alternative quantifications of pitch weighting. Our approach gives equal weight to each note regardless of the note’s harmonic or melodic function. In principle it would be possible to explore the effect of alternate weighting systems sensitive to harmonic context and musical texture, which would be informative as prior research has illustrated that thicker textures (i.e., harmonized music as opposed to non-harmonized) are perceived as “sadder” than thinner textures (Webster and Weir, 2005). Moving to an automated process for cue extraction/re-analysis would be helpful toward that goal. Additionally, it might also be profitable to explore the use of other cues shared with speech (such as timbre) to examine further potential similarities between the two domains. Regardless of the specific topic, these 24 prelude sets serve as an intriguing corpus for such analyses.

Finally, our results suggest potential value in examining the use of these cues across the scope of musical history and geography to see how innate evolutionary/physiological influences and cultural pressures interact to shape music’s historical evolution. Research in empirical musicology shows broad and systematic changes across different musical eras (Turner and Huron, 2008; Post and Huron, 2009; Horn and Huron, 2015) and differences between clearly delineated linguistic cultures (Patel and Daniele, 2003a). Therefore, future analysis of longitudinal changes based on Beuerman’s (2003) compilation of 24-piece prelude compositions written between the late Renaissance era to the end of the 20th century could prove informative. This listing includes 46 individual sets (classifying preludes and fugues separately), which could in theory serve as a rich and “balanced” corpus for a large-scale analytical exploration. The ability to explore shifts in the use of cues such as pitch and timing over periods spanning centuries provides an interesting complement to existing corpus analyses of speech by offering insight into ways in which patterns of communication change over periods of time pre-dating the creation of recording devices.

## Conflict of Interest Statement

The authors declare that the research was conducted in the absence of any commercial or financial relationships that could be construed as a potential conflict of interest.

## Footnotes

1 In music, mode refers to a systematic organization of pitches and the relationship between them. Two of the most commonly used modes in Western music are the major and minor.

2 Although the exact output of these composers is the subject of some debate amongst musicologists, the number of disputed pieces is small relative to their total body of work.

3 Henceforth, we will use Chopin’s *Preludes* to refer to Chopin’s Preludes op. 28, Bach’s *Preludes* to refer to the preludes of the Well-Tempered Clavier, book 1, and Bach’s *Fugues* to refer to the fugues from the Well-Tempered Clavier, book 1.

4 Palmer refers to these tempo markings as the “Kohler tempos.”

5 In Chopin’s *Preludes*, there were two pieces with pick-up measures in the major-key preludes, and three in the minor-key preludes. In the *Well-Tempered Clavier*, only the F major fugue featured pick-up measures.

6 For the sake of consistency we used this same duration numbering scheme regardless of the time signature (i.e., quarter notes received a value of 1 whether they represented a beat in a 4/4 measure or two thirds of a beat in a 6/8 measure).

7 Admittedly, ornamentation in the Baroque era is a complicated system, including a number of unmarked conventions (including cases where appoggiaturas are occasionally played *before* the beat (Wollitz, 1966). While these conventions are clearly musically important, it is not likely to significantly change our absolutely gross measure of average pitch height, and almost certainly did not affect our comparisons between the major and minor key averages (grace notes appeared in 4% of the major-key pieces vs. 3% of the minor-key pieces). Therefore, for the sake of consistency we used these assumptions for coding all ornaments.

8 Although each of the voices in a fugue can be thought of as a separate instrument, in the event when two voices cross, physical limitations of the keyboard dictate that both voices would be physically restricted to sounding for the same duration, even if notated differently.

9 Although “articulation rate” is used in speech research, the musical term *articulation* that refers to the connection between notes rather than the their speed. Consequently we chose to use the term “attack rate” in this context, which serves as a musical parallel of speech articulation rate.

10 Although it is possible that one of the grace notes in **Figure 2** would line up with one of the attacks in the right hand in some performances, the difference between geographical, historical, and even personal performance practice made it impossible to quantify the exact placement of the grace notes. Therefore we counted each grace note as an attack to be consistent.

11 Performed using the ols package (version 4.2-1) in [R Core Team (2014). R: A language and environment for statistical computing. R Foundation for Statistical Computing, Vienna, Austria. URL http://www.R-project.org/].

12 Bartok omitted tempo information for Bach’s *Prelude* 17 and *Fugue* 17 (Ab Major), as well as his *Prelude* 23 and *Fugue* 23 (B Major). We used the average of the other six editors’ recommendations for those pieces as a “replacement” for those otherwise undefined values.

## Supplementary Material

The Supplementary Material for this article, Data Sheet 1, can be found online at: http://journal.frontiersin.org/article/10.3389/fpsyg.2015.01419, and at the MAPLE Lab Tempo Visualizer for JS Bach’s *Well Tempered Clavier* at: http://maplelab.net/bachTempi
