# Supplementary material for: Cueing musical emotions: An empirical analysis of 24-piece sets by Bach and Chopin documents parallels with emotional speech
Source: Front Psychol. 2015 Nov 2;6:1419. doi: 10.3389/fpsyg.2015.01419 (PMC4629484; doi:10.3389/fpsyg.2015.01419)
Supplement: Supplementary file 1 [file Data_Sheet_1.DOCX]

Appendix

Challenging notational issues

|  | **Corpora** | | | | | | **Percent of Total** | |
| --- | --- | --- | --- | --- | --- | --- | --- | --- |
|  | Bach *Preludes* | | Bach *Fugues* | | Chopin *Preludes* | |  |  |
|  | Major | Minor | Major | Minor | Major | Minor | Major | Minor |
| Grace notes |  | 6 |  |  | 11 | 3 | 3.8% | 3.1% |
| Ornaments | 8 | 14 | 8 | 8 | 4 | 1 | 6.9% | 8.0% |
| Arppegiata |  | 7 |  |  | 2 | 4 | 0.7% | 3.8% |
| Double-stemmed notes | 2 | 8 | 6 | 6 | 11 | 14 | 7% | 10% |

**Table A1:** Exceptions encountered during the analysis, separated by each of the three corpora and by mode. The right-most two columns show the exceptions as a percentage of total number of measures (288 measures from 36 pieces for both major and minor keys).
